# Quantifying heterogeneity in SARS-CoV-2 transmission during the lockdown in India

**DOI:** 10.1101/2020.09.10.20190017

**Published:** 2020-09-15

**Authors:** Nimalan Arinaminpathy, Jishnu Das, Tyler H. McCormick, Partha Mukhopadhyay, Neelanjan Sircar

**Affiliations:** 1 MRC Centre for Global Infectious Disease Analysis, Imperial College School of Public Health; 2 McCourt School of Public Policy and the Walsh School of Foreign Service, Georgetown University; 3 Departments of Statistics and Sociology, University of Washington; 4 Centre for Policy Research, New Delhi, India; 5 Ashoka University, Sonipat, India

## Abstract

The novel SARS-CoV-2 virus shows marked heterogeneity in its transmission. Here, we used data collected from contact tracing during the lockdown in Punjab, a major state in India, to quantify this heterogeneity, and to examine implications for transmission dynamics. We found evidence of heterogeneity acting at multiple levels: in the number of potentially infectious contacts per index case, and in the per-contact risk of infection. Incorporating these findings in simple mathematical models of disease transmission reveals that these heterogeneities act in combination to strongly influence transmission dynamics. Standard approaches, such as representing heterogeneity through secondary case distributions, could be biased by neglecting these underlying interactions between heterogeneities. We discuss implications for policy, and for more efficient contact tracing in resource-constrained settings such as India. Our results highlight how contact tracing, an important public health measure, can also provide important insights into epidemic spread and control.

## Introduction

There is increasing recognition of pronounced heterogeneity in the transmission of SARS-CoV-2: that is, that the majority of transmission events appear to be caused only by a small proportion of infected individuals (1–4). Previous modelling work has highlighted the importance of heterogeneity in the emergence of novel pathogens (5), as well as its implications for herd immunity to SARS-CoV-2 (3, 6). Understanding heterogeneity can also have important implications for control, if interventions can be targeted at those most likely to contribute to transmission (7). The need to streamline resources in this way is especially pressing in low- and middle-income settings, given fears that healthcare services in these settings would be particularly challenged by SARS-CoV-2 (8). Here, we analysed data collected from contact tracing during the lockdown in Punjab, a major Indian state, to understand heterogeneity of transmission in this setting, and its implications for control.

## Epidemiological context

**Figure 1:**
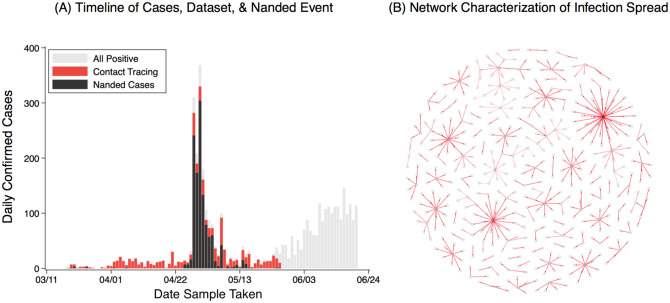
The data from Punjab. (A) Timeseries of reported cases in Punjab during the period of lockdown in the state (red bars) and those due to the Nanded event (black bars), and total cases from early March to the middle of June. (B) Visualisation of case clusters in the dataset, and their linkages from self-reported contacts. This network-type graph requires assumptions (see Materials and Methods). Most individuals infected only few others, while a few infected many: overall, 10% of cases accounted for 80% of infection events.

Punjab, a state in India of about 30 million inhabitants, went into lockdown from 1st April to May 26th (Fig.1A). As elsewhere in India, the lockdown heavily restricted the movement of populations, in most cases to their homes and immediate neighbor-hoods. Travelling outside the house required a special pass, except for essential activities which were also restricted to certain times of the day. The Government of Punjab conducted intensive contact tracing during this time, amongst all known contacts of positive cases, and regardless of symptom status. Due to the ease of tracking individuals during the lockdown, 95% of high-risk contacts (defined as those having face-to-face conversation for at least 15 minutes) could be effectively traced and tested. Overall, this data constitutes the census of all infected persons and their contacts in the state; owing to the lockdown conditions, it affords a unique opportunity to measure contacts with greater accuracy than would be possible during normal economic activity.

The data includes 454 initial cases and 11309 high risk contacts (Fig.1B). Confirmed cases comprise two groups: those residing in Punjab and who were likely infected within the state, and those who are thought to have acquired infection outside the state, due to travel or migration. Our analysis focuses on the former group, and in particular on *seeds* (the first infection in a cluster) in this group, these being the individuals amongst whom contacts are most clearly defined (see Materials and Methods). This yields a total of 148 seeds with 2763 contacts, although we also present sensitivity analysis when analysing all 454 seeds with at least one contact (and all 11309 contacts) in this data, a significant proportion (36%) of whom were religious pilgrims who returned to Punjab from Nanded, Maharashtra, after being stranded there for a month.

## Heterogeneity in transmission

**Figure 2:**
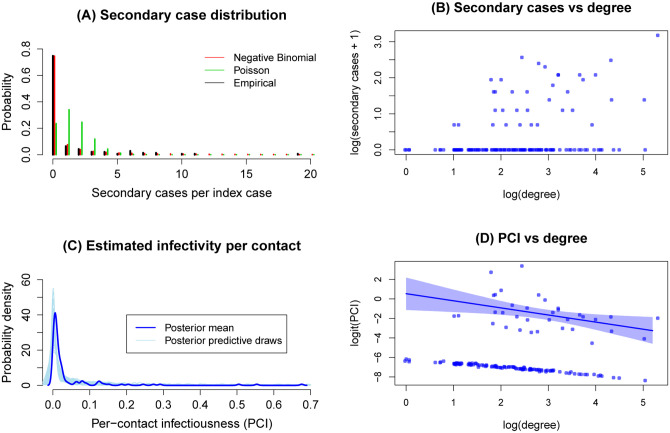
Heterogeneity of the data in secondary cases, and in numbers of contacts. (A) The distribution of secondary cases amongst ‘seeds’ (i.e. first cases in each cluster shown in Fig. 1B). Also shown, for comparison, are the best-fitting Poisson distribution (with *λ* = 1.4)), and the best-fitting negative binomial distribution (with distribution parameters *r* = 0.067, *k* = 0.1). The difference between the latter two curves illustrates the strong extra-Poisson variation in the secondary case distribution. (B) Scatter plot of secondary cases vs degree, at the individual level. The secondary case and degree distributions are shown at the logarithmic scale, and adjusted by 1 to account for zeros, to address skewness of the distributions. Although both secondary case and degree distributions show a strong right-skew (panel A), this figure illustrates that the latter does not explain the former: despite a positive relationship between the two distributions, a substantial number of individuals with low degree generate some infections, while many with high degree generate zero onward infections. (C) Estimated marginal density of per-contact-infectiousness (PCI) that, alongside degree, is needed to explain the heterogeneity in secondary cases. Shaded intervals show 95% Bayesian credibility intervals. (D) Estimated PCI vs degree. The figure displays relationship between the logarithm of the odds (logit) of PCI and the logarithm of the degree. These transformations allow us to plausibly model the joint distribution of PCI and degree as a multivariate normal in section 4 (see Materials and Methods and Supporting Information). There is a discernible lower band due to a large number of cases with zero onward infections, which have very low estimated PCI. Among those with onward infections, there is a discernible negative association.

The “secondary case distribution” is the distribution for the number of onward infections caused by an infected individual. We observe both the number of secondary cases for each individual, and the total number of contacts the person has. In mathematical modelling of transmission dynamics, heterogeneity in transmission is conventionally captured through modelling the secondary case distribution with a negative binomial distribution, allowing for extra-Poisson variation (1, 5). Fig. 2A illustrates the secondary case distribution in the data from Punjab. An important feature in this distribution, consistent with earlier findings (4), is that the majority (76%) of infected cases shows no evidence of onward transmission amongst any of their contacts. The negative binomial distribution captures these individuals, as well as the right-hand tail of the distribution, for example the 10% of individuals accounting for about 80% of transmission in this data. However, this distribution conceals further levels of heterogeneity, that can be important for epidemiological outcomes.

Heterogeneity in transmission can arise from both biological and behavioural factors, including connectedness (the individuals with the most contacts having the most opportunities for transmission), and individual-level variation in infectiousness (for example, with between-individual and temporal variation in viral shedding (9, 10)). Fig. S2 (supporting information) illustrates the distribution in the number of reported contacts per infected case (the ‘degree distribution’) in our dataset, showing a pronounced right-skew similar to that of the secondary case distribution. However, this skew alone cannot explain the heterogeneity in the secondary case distribution: Fig.2B shows that there are many individuals in this data set who caused no further infections despite having many contacts (i.e. having ‘high degree’), and conversely many individuals with low- and moderate-degree who caused several onward infections. These data suggest that there is further heterogeneity acting at the individual level, modifying the effect of the degree distribution (see also Fig. S3).

To capture this heterogeneity we defined the ‘per-contact infectiousness’ (PCI) as the probability that a given contact results in infection, a probability assumed to vary by index case, but to apply equally to all contacts of a given index case. As shown in Fig.2B, there are several individuals with 1–2 contacts who caused zero onward infections, giving rise to substantial uncertainty in their true PCI (similar challenges apply to low-degree individuals who infected all their contacts). To address this issue we treated PCI as an individual-level effect and estimated it using Bayesian shrinkage, a technique employed (among other places) in the education statistics literature to estimate teacher effectiveness (11–13). Fig.2C shows resulting estimates for the marginal distribution of PCI over the population, once again illustrating a strong right-skew. Fig.2D illustrates this association between degree and PCI, showing: (i) a bimodal relationship between the two, arising from the large proportion of individuals that do not infect any others, and (ii) amongst those that do infect others, a negative association between degree and PCI. Overall, these findings illustrate that degree and PCI operate in tandem to drive heterogeneity in the secondary case distribution. Performing these analyses on the full data for seeds (including returnees as well as the ‘core’ group) shows qualitatively similar results (see Fig. S4). We next examined the implications of these associations, for transmission dynamics.

## Implications for transmission dynamics

We asked: (i) how important are the zero-infectors in these distributions, for epidemiological dynamics? (ii) How do outbreak dynamics compare when taking the conventional approach of using the secondary case distribution alone (Fig. 2A) vs when modelling both PCI and degree separately (Fig. 2D)? To address (i), we used the Poisson and negative binomial distributions shown in Fig.2A, the former being an example of capturing the mean secondary cases but failing to capture the proportion zero-infectors. To address (ii), we additionally modelled the log-transformed degree and logit-transformed PCI as following a bivariate normal distribution, with correlation *ρ* (see Table 1, and Materials and Methods). Consistent with Fig.2D, we assumed a range of values for *ρ*, from −0.4 to 0.

For the transmission model we implemented a simple network simulation, in an assumed population of 3,000 individuals, consistent with the population size in this study. For simplicity and generality, we simulated the epidemic in generations of infection: our simulated outbreak behaviour would thus apply to any emerging infection sharing these heterogeneities (see Materials and Methods).

**Figure 3:**
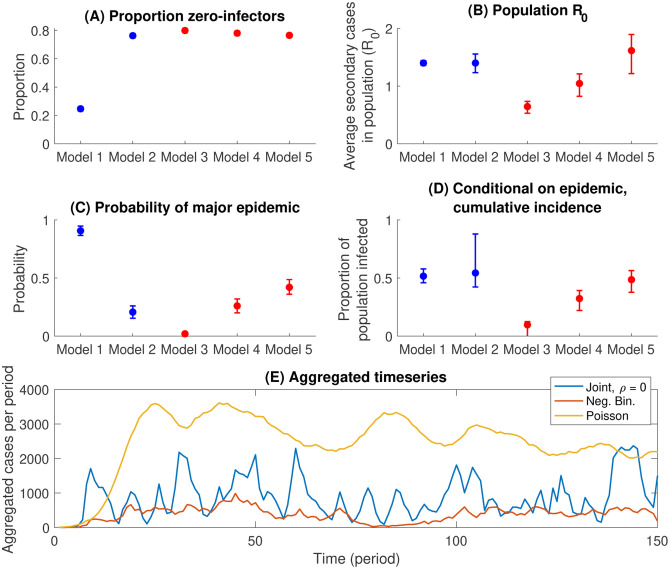
Results of simple transmission models incorporating heterogeneity. Top panels show the average behaviour of an index case in a fully susceptible population of 3,000: (A) The proportion of individuals that cause no further infections. (B) Distributions for the mean number of secondary cases caused by an index case, when averaged over the whole population. In each panel, blue points show outcomes when simulating only secondary case distributions, while red points show outcomes when simulating from the joint degree/PCI distribution described in the main text. Model numbers are as listed in Table 1. Of all models, only the negative binomial secondary case distribution, and the joint degree/PCI models capture the high proportion of index cases who do not cause secondary cases (panel A). However, even amongst these models, there can be substantial variation in *R*_0_ (panel B), owing to the role of correlation between degree and PCI. Middle panels (C,D) show epidemic outcomes over 500 time periods, assuming a 1% probability per time period, of exogenous introduction of an infectious case (here, an ‘epidemic’ is denoted as any simulation having a cumulative incidence > 500 cases (see Materials and Methods for rationale)) . Uncertainty intervals arise from repeating simulations 250 times, and reflect 95% simulation intervals. (E) Modelled timecourse of incidence, when aggregated over 250 simulations (with each simulation being interpreted here as an independent location). A Poisson secondary case distribution (in yellow) gives rise to a large surge in aggregate infection because of epidemics in multiple locations occurring in a synchronised way.

Fig. 3 shows a comparison of model projections for the behaviour of an index case: that is, when simulating only a first generation of infection. Results illustrate how it is possible to accommodate a wide range for *R*_0_ (Fig. 3A), even amongst models that capture a high proportion non-infectors (Fig. 3B).

Figs. 3C,D compare the outcomes of full epidemic simulations. By failing to capture the high proportion of zero-infectors (Fig. 3A), a Poisson secondary case distribution yields the most outbreak-prone populations, with 90% of simulations yielding major epidemics (Fig. 3C). Even amongst the remaining models, however, there is a notable disparity in epidemiological outcomes: amongst models capturing the joint distribution between degree and PCI (Models 3 – 5), it is not possible to identify a value of *ρ* that matches most closely to the negative binomial model for secondary cases (Model 2). While the latter appears intermediate to Models 3 and 4 in Fig. 2C, it is intermediate to Models 4 and 5 in Fig. 2D.

Figure 3E compares selected models in terms of the aggregate temporal pattern that they predict, when aggregated over multiple independent locations. Under epidemics simulated using a Poisson secondary case distribution, there is a surge of infection across several locations at once, a scenario that would place severe demands on health resources. By contrast in outbreaks driven by distributions capturing the high proportion zero-infectors, aggregate epidemic dynamics are more characterised by a series of asynchronous peaks in different locations, overall making for a lower peak demand on health services.

## Efficiency of contact tracing

**Figure 4:**
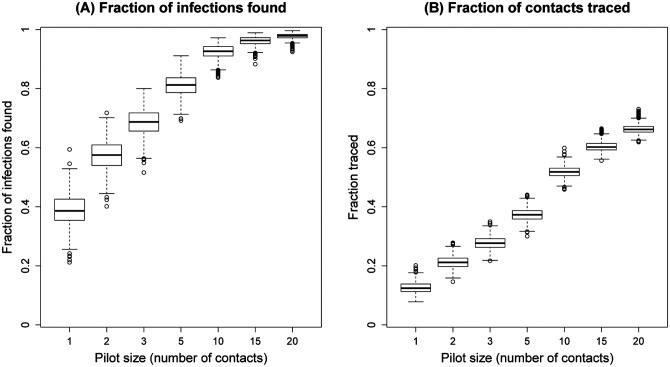
An approach to efficient contact tracing. Figure shows simulated outcomes of a strategy to test all contacts of an index case, only if there is at least one positive individual in an initial ‘pilot’ sample of *s* contacts. (A) The proportion of infections found as a function of *s* (B) Overall contact tracing effort, as measured by the proportion of contacts that would be traced, again as a function of *s*. Owing to the right-skew of the PCI, the left-hand panel illustrates diminishing returns with increasing *s*, suggesting, for example, that it would be possible to identify 80% of the cases in this dataset, with <40% of the contact tracing effort.

Although contact tracing plays an important role in the SARS-CoV-2 response, in resource-constrained settings such as India, its demands on the healthcare system can make it difficult to sustain. Motivated by our findings, we propose reframing contact tracing with the goal of efficiently identifying individuals with high PCI. In our data overall, we estimate that if an individual caused at least one onward infection, there is a 79% probability that they caused at least two onward infections. We thus propose a sequential strategy where, for every index case, a ‘pilot’ subset of only *s* randomly selected contacts is first tested; the remainder of contacts are then followed up and tested, only if there is a positive in the pilot subset. Such a strategy could substantially reduce the overall contact tracing effort, while still effectively identifying high PCI individuals. Fig. 4 shows results of simulating such a strategy 1,000 times on the full dataset of 454 cases, for a range of values of *s*. The figure illustrates diminishing returns in the fraction of infections found, beyond a pilot subset size of 10 contacts (Fig. 4A). However, even with a pilot subset size of only 5 contacts, it is possible to identify 80% of infections (Fig. 4A), with <40% of the contact tracing effort that was expended in this data (Fig. 4B).

## Discussion

We have shown how individual-level data, gathered from the routine course of contact tracing, can be analysed to gain important insights into the transmission of SARS-CoV-2. As well as affirming findings from elsewhere, that the majority of cases appear not to infect any others (1, 4), our findings also highlight how heterogeneity in transmission may be more complex than previously recognised.

Simple dynamical models highlight the important role that is played by these heterogeneities. At the gross level of the secondary case distribution, the high proportion of zero-infectors yields outbreak dynamics wherein surges can be handled by providing mobile services rather than increasing hospital capacity in every geography (Fig. 3E). The negative binomial distribution, conventionally used in the modelling literature, captures this proportion well (Fig. 2A). However, our analysis also highlight some limitations of this distribution: accounting for underlying correlations between degree and PCI can lead to different outbreak dynamics, in terms of the risk and size of major outbreaks over time (Figs. 3C,D). Our results also have implications for the efficiency of contact tracing. When a large fraction of infected individuals do not cause onward transmission, we show the value of a simple two-step strategy, of, for example, first testing family members and then testing other contacts only if at least one family member is found to be positive (Fig. 4). Such approaches can be particularly valuable in resource-constrained settings such as India, in decreasing the requirements for contact tracing substantially, while still identifying most cases.

An important question, that we are not able to address using the current data, is what drives the heterogeneity in per-contact infectiousness. This heterogeneity may arise, for example, from biological factors such as the role of pre-existing, cross-reactive immunity that may moderate viral load in some individuals more effectively than others (14). Our analysis suggests that PCI increases with age and is significantly associated with sex (Fig. S5). Further data on these and other individual-level characteristics would be invaluable in further examining key risk factors for infectiousness. Where risk factors involve individual characteristics that can be readily identified in newly diagnosed patients, such as viral load, these factors could also play an important role in guiding future contact tracing efforts. However, heterogeneity in PCI could also reflect variations in the closeness of reported contacts, with some reporting only the closest contacts and others reporting wider contacts, thus explaining the negative correlation. We emphasise that our data is limited to defined ‘high-risk’ contacts (see Materials and Methods), thus excluding incidental contacts that might be expected to bias our estimates the most. Nonetheless, even if there is variation in closeness amongst high-risk contacts, our analysis offers an approach for adjusting for these variations, when interpreting what routine contact tracing data means for transmission: in this case our estimates for PCI should be regarded as a data-driven weighting of contacts, rather than infectiousness. Our approach can easily be adapted for any dataset where there is additional information on closeness of contact.

Amongst other limitations, the contact tracing data was collected, not under controlled study conditions, but as part of a public health response, by the Government of Punjab. Our approach to this data is pragmatic, recognising some inherent limitations: there may be false negatives in the data if people were tested too early, or indeed if people had been infected long in the past, which we cannot tell in the absence of serological tests. As with any contact tracing data, our assumptions for who-infected-whom, in a given contact pair, may be imperfect. We are able to address some of these concerns (for instance, by showing that our results are robust to a change in the directionality of a link [see Materials and Methods]). However, more–and better–data are absolutely necessary to refine our estimates, particularly on the nature of the correlation between degree and PCI. Further, although the lockdown conditions facilitate an in-depth analysis of transmission amongst contacts, our findings must be interpreted with caution in scenarios with uninhibited transmission, as might occur in the absence of a lockdown or other non-pharmaceutical interventions (15). Additional limitations on the modelling are described in the Materials and Methods.

Overall, the methods that we have outlined here should apply to any contact tracing database and our publicly available code can be directly applied to any such data that have been collected. Contact tracing forms an integral part of the response to SARS-CoV-2 around the world: while being an important public health strategy in its own right, it can also provide invaluable information about how, and to whom, infection is being spread. Systematic analysis of this data could provide important insights to inform future, smarter strategies for the control of SARS-CoV-2.

## Supplementary Material

1

## Figures and Tables

**Table 1:** List of the different models used, for capturing heterogeneity in the population. ‘Secondary case distributions’ (models 1 – 2) are as in Fig. 2A. They ignore any interactions between degree and PCI, and instead aim only to capture variation in the numbers of secondary cases per index case. By contrast, ‘Joint distributions’ aim to model the associations shown in Fig. 2D. They employ the bivariate normal distribution described in the Materials and Methods, with correlation *ρ*.

Model number	Description
1	Secondary case distribution using Poisson distribution with mean 1.4
2	Secondary case distribution using Negative Binomial distribution with number of successes = 0.1 and probability of success = 0.067
3	Joint degree/PCI distribution with *ρ* = −0.4
4	Joint degree/PCI distribution with *ρ* = −0.2
5	Joint degree/PCI distribution with *ρ* = 0

## References

[1] Endo A., Centre for the Mathematical Modelling of Infectious Diseases COVID-19 Working Group, Abbott S., Kucharski A. J., Funk S., Wellcome Open Research 5, 67 (2020). 32685698 10.12688/wellcomeopenres.15842.1 PMC7338915

[2] Wang Y., Teunis P., Frontiers in Medicine 7, 329 (2020). 32637423 10.3389/fmed.2020.00329 PMC7317008

[3] Gomes M. G. M., , MedRxiv p. 2020.04.27.20081893 (2020).

[4] Laxminarayan R., , medRxiv (2020).

[5] Lloyd-Smith J. O., Schreiber S. J., Kopp P. E., Getz W. M., Nature 438, 355 (2005). 16292310 10.1038/nature04153 PMC7094981

[6] Britton T., Ball F., Trapman P., Science 369, 846 (2020). 32576668 10.1126/science.abc6810 PMC7331793

[7] Wallinga J., van Boven M., Lipsitch M., Proceedings of the National Academy of Sciences 107, 923 (2010). 10.1073/pnas.0908491107 PMC2818907 20080777

[8] Walker P. G. T., , Science 369, 413 (2020).32532802

[9] Fu Y., , European Respiratory Journal (2020).

[10] Qi L., , International Journal of Infectious Diseases 96, 531 (2020). 32425636 10.1016/j.ijid.2020.05.045 PMC7231495

[11] Mendro R., , Annual Meeting of the American Educational Research Association, San Diego, CA (1998).

[12] Lockwood J., , Journal of Educational Measurement 44, 47 (2007).

[13] Lockwood J., McCaffrey D. F., Mariano L. T., Setodji C., Journal of Educational and Behavioral Statistics 32, 125 (2007).

[14] Le Bert N., , Nature (2020).

[15] Report 9: Impact of non-pharmaceutical interventions (npis.

[16] Ministry of Health & Family Welfare, India, Guidance document for POEs, states and UTs for surveillance of 2019-nCoV, Tech. rep. (2020).

[17] Gelman A., , Bayesian Data Analysis, Third Edition, Chapman & Hall/CRC Texts in Statistical Science (Taylor & Francis, 2013).

[18] Gelman A., Hill J., Data Analysis Using Regression and Multilevel/Hierarchical Models (Cambridge University Press, 2006).

[19] Verity R., , The Lancet Infectious Diseases (2020). 10.1016/S1473-3099(20)30706-4 PMC7837005 32919520

[20] Liu Y., , The European respiratory journal 55, 2001112 (2020). 32269087 10.1183/13993003.00697-2020 PMC7144267

[21] Danon L., , Interdisciplinary perspectives on infectious diseases 2011, 284909 (2011). 21437001 10.1155/2011/284909 PMC3062985

